# Symptom Profiles Compatible With Disorders of Gut‐Brain Interaction in Individuals With Coeliac Disease on a Long‐Term Gluten‐Free Diet

**DOI:** 10.1002/ueg2.70311

**Published:** 2026-10-16

**Authors:** Tom van Gils, Hans Törnblom, Floris van Overveld, Olafur S. Palsson, Ami D. Sperber, Shrikant I. Bangdiwala, Inês A. Trindade, Marc A. Benninga, Gerd Bouma, Magnus Simrén

**Affiliations:** ^1^ Department of Molecular and Clinical Medicine Institute of Medicine Sahlgrenska Academy University of Gothenburg Gothenburg Sweden; ^2^ Department of Medicine Sahlgrenska University Hospital Gothenburg Region Västra Götaland Sweden; ^3^ Dutch Coeliac Society Naarden the Netherlands; ^4^ University of North Carolina/Chapel Hill Chapel Hill North Carolina USA; ^5^ Faculty of Health Sciences Ben‐Gurion University of the Negev Beer‐Sheva Israel; ^6^ Department of Health Research Methods, Evidence and Impact McMaster University Hamilton Ontario Canada; ^7^ Population Health Research Institute McMaster University Hamilton Ontario Canada; ^8^ EMBRACE Lab Center for Health and Medical Psychology Örebro University Örebro Sweden; ^9^ Department of Paediatric Gastroenterology Emma Children's Hospital Amsterdam University Medical Centers University of Amsterdam Amsterdam the Netherlands; ^10^ Amsterdam Gastroenterology Endocrinology Metabolism Amsterdam the Netherlands; ^11^ Department of Gastroenterology and Hepatology Amsterdam University Medical Centers Amsterdam the Netherlands

**Keywords:** coeliac disease, disorders of gut‐brain interaction, epidemiology, irritable bowel syndrome, quality of life

## Abstract

**Background:**

Persistent gastrointestinal (GI) symptoms are frequently reported in patients with coeliac disease (CD). Knowledge about the role of disorders of gut‐brain interaction (DGBI) as the cause of these persistent symptoms remains limited.

**Objective:**

To assess the prevalence and factors associated with DGBI‐compatible symptom profiles in individuals with CD on a long‐term gluten‐free diet (GFD).

**Methods:**

Adult members of the Dutch Coeliac Society completed an online survey, including the Rome IV diagnostic questionnaire and validated questionnaires assessing psychological distress, non‐GI somatic symptom severity, and health‐related quality of life (QOL). Only individuals with CD adhering to a GFD ≥ 1 year were included. A population‐based Dutch cohort served as the reference group.

**Results:**

Among 1001 participants with CD (79.3% female; median age 50 years), 64.8% met the criteria for ≥ 1 DGBI‐compatible symptom profile compared with 31.3% in the reference group (for confounders adjusted odds ratio (AOR) 3.2 (95% CI 2.6–3.8)). Irritable bowel syndrome showed the strongest association with CD (AOR 4.5 (95% CI 3.3–6.2)). Having DGBI‐compatible symptom profiles was, like the reference group, associated with higher levels of psychological distress and non‐GI somatic symptom severity and lower health‐related QOL.

**Conclusions:**

Rome IV DGBI‐compatible symptom profiles are highly prevalent in individuals with CD on a long‐term GFD and are associated with impaired QOL. While ongoing gluten exposure and other comorbidities could not be excluded, these findings support the consideration of DGBI in the evaluation and treatment of persistent symptoms in patients with CD.

## Key Summary

- Summarize the established knowledge on this topic
  ◦ Persistent symptoms in patients with coeliac disease on a long‐term gluten‐free diet are common.
  ◦ Symptoms compatible with irritable bowel syndrome (IBS) are common in individuals with coeliac disease on a long‐term gluten‐free diet. However, data about the prevalence of other disorders of gut‐brain interaction (DGBI) are sparse.
- What are the significant and/or new findings of this study?
  ◦ DGBI‐compatible symptom profiles are more common in individuals with coeliac disease on a long‐term gluten‐free diet compared to the general population.
  ◦ IBS is the most common DGBI in individuals with coeliac disease on a long‐term gluten‐free diet.
  ◦ Similar factors associated with DGBI in the general population are associated with DGBI‐compatible symptom profiles in patients with coeliac disease, including female sex, younger age, psychological distress, and lower quality of life.

## Graphical abstract

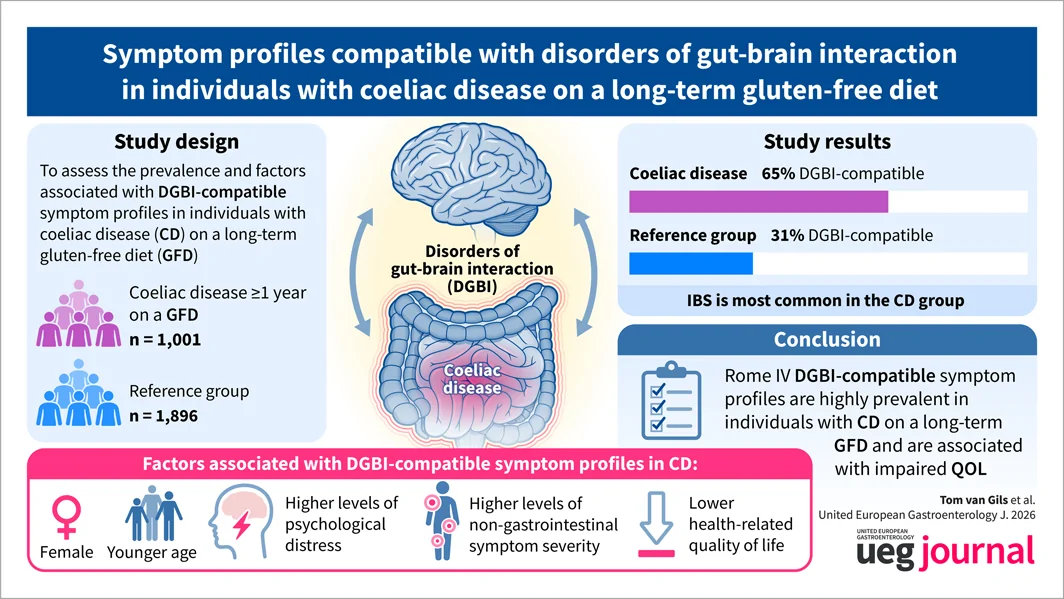

## 1 Introduction

Coeliac disease (CD) is a chronic immune‐mediated enteropathy, triggered by the ingestion of gluten [1]. The estimated global prevalence is around 1%, with a female predominance [2]. The diagnosis is based on a combination of the presence of CD‐specific auto‐antibodies and histological abnormalities in duodenal biopsies, with a strict life‐long gluten‐free diet (GFD) being the only currently available treatment strategy [1, 3]. Based on the most recent European paediatric and adult guidelines, histological assessment can be omitted in specific diagnostic situations [1, 4].

Persistent symptoms in CD patients on a long‐term GFD are common [5, 6, 7]. A recent meta‐analysis showed that the prevalence of non‐responsive CD, defined as persistent clinical manifestations despite a ≥ 6 months GFD, varied between 12.5% and 50% [8]. One of the potential causes of persistent symptoms are disorders of gut‐brain interaction (DGBI), formerly known as functional gastrointestinal (GI) disorders, currently diagnosed according to the Rome V criteria [9, 10]. DGBI, for example irritable bowel syndrome (IBS), are symptom‐based GI disorders with a global prevalence of 40.3% (Rome IV) or 42.2% (Rome V), depending on the criteria used [11, 12]. Underlying pathophysiological mechanisms are complex, including altered GI sensorimotor function, mucosal immune activity, gut microbiome, and central nervous system processing of gut signalling [13].

According to the definition of DGBI, these conditions cannot be diagnosed when an organic disease explains the symptoms [13]. However, there is consensus that a DGBI can co‐exist with an organic GI disease in remission, although the diagnostic assessment can be challenging [14]. Only a few studies have investigated DGBI in patients with CD on a long‐term GFD [9]. Most studies focused on IBS only, often lacked a control group, or studied a paediatric population [15]. Therefore, the aim of this study was (1) to investigate the prevalence of DGBI‐compatible symptom profiles in adult patients with CD on a long‐term GFD, (2) to assess whether these symptom profiles are more prevalent in the CD group vs. the general population, and (3) to assess factors associated with DGBI‐compatible symptom profiles in patients with CD on a long‐term GFD.

## 2 Materials and Methods

### 2.1 Study Setting and Participants

An online survey was sent out by the Dutch Coeliac Society (NCV) in June and July 2025. This was done via the NCV website and social media accounts, as well as via a newsletter sent to NCV members. The NCV is the Dutch patient society for individuals with gluten‐related diseases and disorders with 16,119 adult members at the time the survey was sent out (age unknown for < 1% of the members). To avoid selection‐bias, participants were told that the aim of the survey was to investigate the health of individuals with CD, rather than a survey about DGBI or persistent GI symptoms. The survey included built‐in quality‐assurance measures, including two repeat questions and one attention question. Only data of individuals with a self‐reported physician‐based diagnosis of CD based on duodenal biopsies (gastroscopy) and/or CD‐specific antibodies (blood test), who reported being ≥ 1 year on a GFD, and who passed the survey quality checks, were included. Individuals who reported a history of a physician‐based diagnosis of inflammatory bowel disease (IBD), GI cancer, diverticulitis, peptic ulcer, or a bowel resection were excluded since these conditions can cause symptoms similar to a DGBI [11].

The reference group consisted of individuals who participated in the Dutch part of the Rome Foundation Global Epidemiology Study (RFGES), with data collected in November and December 2017. The RFGES is a study that investigated the prevalence of all Rome IV 22 DGBI in the general population of 33 countries [11, 16]. For the Dutch part of the RFGES, online surveys were used, including similar quality checks as used in the NCV survey. Individuals with a self‐reported physician‐based CD diagnosis as well as individuals with ≥ 1 of the previously mentioned other five organic conditions were excluded.

Based on the evidence that the prevalence of DGBI may have increased after the COVID‐19 pandemic [17], and because the reference group was recruited pre‐COVID and the CD group post‐COVID, we performed sensitivity analyses using a second reference group (‘NCV reference group’). This NCV reference group included individuals without CD who were recruited by CD participants who completed the survey and who forwarded the survey to non‐CD acquaintances. The NCV reference group was not used as the primary reference group because of the risk of selection bias (the surveys were forwarded by CD participants) and because the NCV reference group was small, which would not allow us to perform analyses for most of the individual DGBI.

More details about the selection of individuals are shown in the study flow chart (Supporting Information S1: Figure S1).

### 2.2 Questionnaires

The survey included validated questionnaires comprising 178 questions. However, because of built‐in skip patterns, most participants completed fewer questions. Because the Rome V diagnostic questionnaire, which was published in 2026, was not yet available at the time of the study, the Dutch translation of the Rome IV diagnostic questionnaire was used to assess the presence of all 22 DGBI‐compatible symptom profiles [18]. Furthermore, the PHQ‐12 (non‐GI somatic symptom severity [19]), PHQ‐4 (psychological distress [20]), and PROMIS‐10 (health‐related quality of life [21, 22]), as well as questions regarding CD diagnosis, medical history, and medical treatment were used. The Coeliac Dietary Adherence Test (CDAT) [23] was initially selected as the primary method for assessing GFD adherence. However, due to poor internal consistency and construct validity of the Dutch CDAT in our cohort, it was not possible to use the CDAT for this purpose. Alternatively, self‐reported adherence to a GFD of ≥ 95% during the last year was used to define optimal GFD adherence for sensitivity analyses. This cut‐off was taken since minor inadvertent lapses less than a few times a year are still considered to be within optimal GFD adherence.

More details about the questionnaires and the translation process and psychometric analyses of the CDAT are described in the Supporting Information S1: Figure S1 and Supporting Information S1: Table S1.

### 2.3 Statistical Methods

Numbers, proportions, medians, and means with 95% confidence intervals (CI) were used for the descriptive analyses. Differences between groups were shown by odds ratios (ORs) or standardized mean differences (SMD), including 95%CI. SMD (Cohen's d) of 0.2–0.5 was considered to reflect small, 0.5–0.8 medium, and > 0.8 large effect sizes [24]. Logistic regression models were used to investigate if the prevalence of DGBI‐compatible symptom profiles (dependent variable) differed between individuals with CD compared to the reference group (independent variable). These associations were presented as crude ORs and adjusted ORs (AOR) after correcting for potential confounding factors, chosen based on clinical knowledge and current literature [25]. There were no signs of strong collinearity in the regression models (variance inflation factor < 2.5 in all models). Sensitivity analyses were performed by including only individuals with CD and an optimal GFD adherence (≥ 95% during the past year) and by using an alternative reference group (NCV reference group).

Based on the explorative character of this study, the use of *p*‐values was avoided [26]. Instead, means, proportions, and effect sizes including 95% CI were presented. Statistical analyses were performed using IBM SPSS (version 29.0.0.0).

### 2.4 Ethics

This study was reviewed by the Dutch medical ethics review committee ‘METC Brabant’ and was determined to be outside the restrictions of the Medical Research Involving Human Subjects Act (WMO) (protocol number: NW2025‐37). The RFGES internet survey was reviewed by the Biomedical Institutional Review Board of the University of North Carolina at Chapel Hill (United States of America) and was deemed exempt from their oversight due to the anonymity of the participants. The study protocols adhere to the ethical guidelines of the Declaration of Helsinki (1975). An electronic consent form was signed by all participants.

## 3 Results

In total, 1001 individuals were included in the CD group, of whom 794 (79.3%) were females with a median age of 50 years (range 18–88 years) (Table 1). The RFGES reference group comprised 1896 individuals, of whom 964 (50.8%) were females, with a median age of 48 years (range 18–88). The characteristics of the NCV reference group, used for sensitivity analyses, are summarized in Supporting Information S1: Table S1

**TABLE 1:** Characteristics of individuals in the CD and reference groups.

	Coeliac disease *n* = 1001	Reference group *n* = 1896
Age, median [IQR]	50 [35–64]	48 [30–62]
Sex		
Female, *n* (%)	794 (79.3%)	964 (50.8%)
Male, *n* (%)	204 (20.4%)	932 (49.2%)
Other, *n* (%)	1 (0.1)	Not available
Unknown, *n* (%)	2 (0.2)	Not available
Completed higher‐level professional or university‐level education, *n* (%)	635 (63.4%)	Not available
Non‐GI somatic symptom severity (PHQ‐12), median [IQR]	6 [3–8]	4 [2–6]
Psychological distress (PHQ‐4), median [IQR]	2 [0–4]	0 [0–3]
Parent, brother, or sister with CD, *n* (%)	229 (22.9%)	31 (1.6%)
CD‐specific		
Diagnosis based on		
Serology and duodenal biopsy, *n* (%)	862 (86.1%)	Not applicable
Serology only, *n* (%)	124 (12.4%)	Not applicable
Biopsy only, *n* (%)	15 (1.5%)	Not applicable
Time on a GFD		
1 year, *n* (%)	47 (4.7%)	Not applicable
2‐4 years, *n* (%)	186 (18.6%)	Not applicable
≥ 5 years, *n* (%)	768 (76.7%)	Not applicable
Age at time of CD diagnosis		
< 18 years	125 (12.5%)	Not applicable
≥ 18 years	798 (79.7%)	Not applicable
Missing data	78 (7.8%)	Not applicable
Self‐reported GFD adherence during the past year		
< 90%, *n* (%)	17 (1.7%)	Not applicable
90%‐95%, *n* (%)	37 (3.7%)	Not applicable
95%‐99%, *n* (%)	270 (27.0%)	Not applicable
100%, *n* (%)	677 (67.6%)	Not applicable

Abbreviations: CD, coeliac disease; GFD, gluten‐free diet; IQR, interquartile range; *n*, number; OR, odds ratio.

### 3.1 Prevalence of DGBI‐Compatible Symptom Profiles in Individuals With CD on a Long‐Term GFD

The prevalence of fulfiling at least one of the 22 Rome IV DGBI‐compatible symptom profiles in the CD group was 64.8% (95% CI 61.8–67.7). The prevalence of any DGBI‐compatible symptom profile was higher in female participants than in male participants (69.4% vs. 47.5%; OR 2.5 (95% CI 1.8–3.4)) and in younger individuals compared to older individuals with CD (18–34 years: 73.6% vs. ≥ 65 years: 55.7%; OR 2.2 (95% CI 1.5–3.3)), Table 2. This difference between the youngest and oldest age groups remained after adjustment for sex (AOR 2.0 (95% CI 1.3–3.0)). No differences were seen for duration of GFD, age at time of CD diagnosis, or level of self‐reported GFD adherence. Similar sex‐ and age‐related differences in the prevalence of DGBI‐compatible symptom profiles were observed in the reference group (Supporting Information S1: Table S2).

**TABLE 2:** The prevalence of any DGBI‐compatible symptom profile in subgroups of individuals with CD on a long‐term GFD.

	Prevalence of having any DGBI‐compatible symptom profile (95% CI)	OR (95% CI)
Total CD group *n* = 1001	64.8 (61.8–67.7)	—
Sex		
Male *n* = 204	47.5 (40.8–54.4)	Reference
Female *n* = 794	69.4 (66.1–72.5)	**2.5 (1.8–3.4)**
Current age		
18–34 years *n* = 242	73.6 (67.6–78.7)	**2.2 (1.5–3.3)**
35–49 years *n* = 257	66.9 (61.0–72.4)	**1.6 (1.1–2.3)**
50–64 years *n* = 272	62.9 (57.0–68.4)	1.3 (0.9–1.9)
≥ 65 years *n* = 230	55.7 (49.2–61.9)	Reference
Time on a GFD		
1 year *n* = 47	61.7 (47.4–74.2)	Reference
2–4 years *n* = 186	66.7 (59.6–73.1)	1.2 (0.6–2.4)
≥ 5 years *n* = 768	64.6 (61.1–67.9)	1.1 (0.6–2.1)
Age at time of CD diagnosis		
< 18 years *n* = 125	62.4 (53.7–70.4)	Reference
18–44 years *n* = 503	68.2 (64.0–72.1)	1.2 (0.9–1.9)
≥ 45 years *n* = 295	59.0 (53.3–64.4)	0.9 (0.6–1.3)
Self‐reported GFD adherence		
< 95% of the time *n* = 54	64.8 (51.4–76.2)	1.0 (0.5–1.8)
95%–99% of the time *n* = 270	63.7 (57.8–69.2)	0.9 (0.7–1.3)
100% of the time *n* = 677	65.3 (61.6–68.8)	Reference

*Note:* Bold values indicate ORs that do not cross one. Three individuals with CD reported to have another sex (*n* = 1) or did not want to report their sex (*n* = 2). Data from *n* = 78 individuals were missing for “age at time of CD diagnosis”.

Abbreviations: CD, coeliac disease; GFD, gluten‐free diet; *n*, number; OR, odds ratio.

Bowel DGBI was most prevalent in the CD group (59.8%), followed by anorectal (12.9%), gastroduodenal (11.2%), and oesophageal (9.3%) (Table 3). Thirty‐three percent of all individuals with a DGBI‐compatible symptom profile met the criteria for DGBI in ≥ 2 anatomical regions (Figure 1A), with the most common overlap observed between bowel and anorectal DGBI (Figure 1B). A similar pattern of overlapping anatomical regions was observed in the reference group, with 27.7% meeting the criteria for DGBI in multiple anatomical regions (Supporting Information S1: Figure 2A–B). Of the individual DGBI, criteria compatible with IBS were most prevalent in individuals with CD (18.2% of individuals with CD), followed by functional constipation (16.2%) and functional bowel disorders (FBD) unspecified (13.6%) (Table 3).

**TABLE 3:** The association between having CD on a long‐term GFD and fulfiling criteria for a Rome IV DGBI.

	Coeliac disease *n* = 1001	Reference group *n* = 1896	Crude OR (95% CI)	AOR (95% CI) Adjusted for sex and age	AOR (95% CI) Adjusted for sex, age, non‐GI somatic symptom severity, and psychological distress
Any DGBI	649 (64.8%)	593 (31.3%)	**4.1 (3.4–4.8)**	**3.6 (3.0–4.3)**	**3.2 (2.6–3.8)**
Any oesophageal DGBI	93 (9.3%)	92 (4.9%)	**2.0 (1.5–2.7)**	**1.8 (1.3–2.5)**	1.3 (1.0–1.9)
Functional heartburn	10 (1.0%)	8 (0.4%)	2.4 (0.9–6.1)	—	—
Functional chest pain	31 (3.1%)	32 (1.7%)	**1.9 (1.1–3.1)**	**1.8 (1.1–3.1)**	1.3 (0.7–2.3)
Reflux hypersensitivity	16 (1.6%)	7 (0.4%)	**4.4 (1.8–10.7)**	—	—
Globus	14 (1.4%)	12 (0.6%)	**2.2 (1.0–4.8)**	—	—
Functional dysphagia	40 (4.0%)	42 (2.2%)	**1.8 (1.2–2.9)**	**1.6 (1.0–2.5)**	1.2 (0.7–1.9)
Any gastroduodenal DGBI	112 (11.2%)	116 (6.1%)	**1.9 (1.5–2.5)**	**1.9 (1.4–2.5)**	**1.4 (1.0–1.9)**
Functional dyspepsia (all subtypes)	71 (7.1%)	78 (4.1%)	**1.8 (1.3–2.5)**	**1.7 (1.2–2.4)**	1.3 (0.9–1.8)
PDS	53 (5.3%)	63 (3.3%)	**1.6 (1.1–2.4)**	**1.6 (1.1–2.3)**	1.1 (0.7–1.7)
EPS	34 (3.4%)	23 (1.2%)	**2.9 (1.7–4.9)**	**2.9 (1.6–5.0)**	**2.2 (1.2–3.9)**
Belching disorder	7 (0.7%)	3 (0.2%)	**4.4 (1.1–17.2)**	—	—
Rumination syndrome	39 (3.9%)	33 (1.7%)	**2.3 (1.4–3.7)**	**2.0 (1.2–3.3)**	1.6 (0.9–2.6)
Chronic nausea and vomiting syndrome	8 (0.8%)	12 (0.6%)	1.3 (0.5–3.1)	—	—
Cyclic vomiting syndrome	7 (0.7%)	7 (0.4%)	1.9 (0.7–5.4)	—	—
Cannabinoid hyperemesis syndrome	0 (0.0%)	0 (0.0%)	—	—	—
Any bowel DGBI	599 (59.8%)	511 (27.0%)	**4.0 (3.4–4.7)**	**3.6 (3.0–4.2)**	**3.2 (2.6–3.8)**
IBS (all subtypes)	182 (18.2%)	73 (3.9%)	**5.5 (4.2–7.4)**	**5.1 (3.8–6.9)**	**4.5 (3.3–6.2)**
*IBS‐C*	50 (5.0%)	25 (1.3%)	**3.9 (2.4–6.4)**	**3.4 (2.1–5.7)**	**2.7 (1.6–4.6)**
*IBS‐D*	60 (6.0%)	25 (1.3%)	**4.8 (3.0–7.7)**	**4.7 (2.8–7.7)**	**3.9 (2.3–6.5)**
IBS‐M	58 (5.8%)	18 (0.9%)	**6.4 (3.8–11.0)**	**5.3 (3.1–9.3)**	**4.3 (2.5–7.6)**
IBS‐U	14 (1.4%)	5 (0.3)	**5.4 (1.9–14.9)**	—	—
Functional constipation	162 (16.2%)	178 (9.4%)	**1.9 (1.5–2.3)**	**1.5 (1.2–2.0)**	**1.4 (1.1–1.8)**
Opioid‐induced constipation	3 (0.3%)	13 (0.7%)	0.4 (0.1–1.5)	—	—
Functional diarrhoea	55 (5.5%)	64 (3.4%)	**1.7 (1.2–2.4)**	**1.5 (1.0–2.2)**	1.3 (0.9–2.0)
Functional abdominal bloating/distension	61 (6.1%)	26 (1.4%)	**4.7 (2.9–7.4)**	**4.2 (2.6–6.8)**	**3.9 (2.4–6.4)**
Unspecified FBD	136 (13.6%)	157 (8.3%)	**1.7 (1.4–2.2)**	**1.6 (1.3–2.1)**	**1.5 (1.2–2.0)**
Any anorectal DGBI	129 (12.9%)	84 (4.4%)	**3.2 (2.4–4.3)**	**2.6 (1.9–3.5)**	**2.1 (1.5–2.8)**
Faecal incontinence	31 (3.1%)	33 (1.7%)	**1.8 (1.1–3.0)**	1.2 (0.7–2.0)	1.0 (0.6–1.7)
Levator ani syndrome	21 (2.1%)	16 (0.8%)	**2.5 (1.3–4.8)**	**2.3 (1.2–4.6)**	—
Proctalgia fugax	86 (8.6%)	40 (2.1%)	**4.4 (3.0–6.4)**	**3.7 (2.5–5.5)**	**2.9 (1.9–4.4)**
Others					
Functional biliary pain	3 (0.3%)	1 (0.1%)	5.7 (0.6–54.8)	—	—
Central abdominal pain syndrome	0 (0.0%)	0 (0.0%)	—	—	—

*Note:* An adjusted odds ratio related to individual DGBI‐compatible symptom profiles was only shown if there were ≥ 10 individuals (CD and reference group combined) with that particular DGBI‐compatible symptom profile per variable included in the multivariable logistic regression model. Bold values indicate ORs that do not cross one.

Abbreviations: AOR; adjusted odds ratio, CD; coeliac disease, DGBI; disorder of gut‐brain interaction, EPS; epigastric pain syndrome, FBD; functional bowel disorder, GFD; gluten‐free diet, IBS; irritable bowel syndrome, IBS‐C; IBS with predominant constipation, IBS‐D; with predominant diarrhoea, IBS‐M; IBS with mixed bowel habits, IBS‐U; IBS unclassified, *n*; number, OR; odds ratio, PDS; postprandial distress syndrome.

**FIGURE 1:**
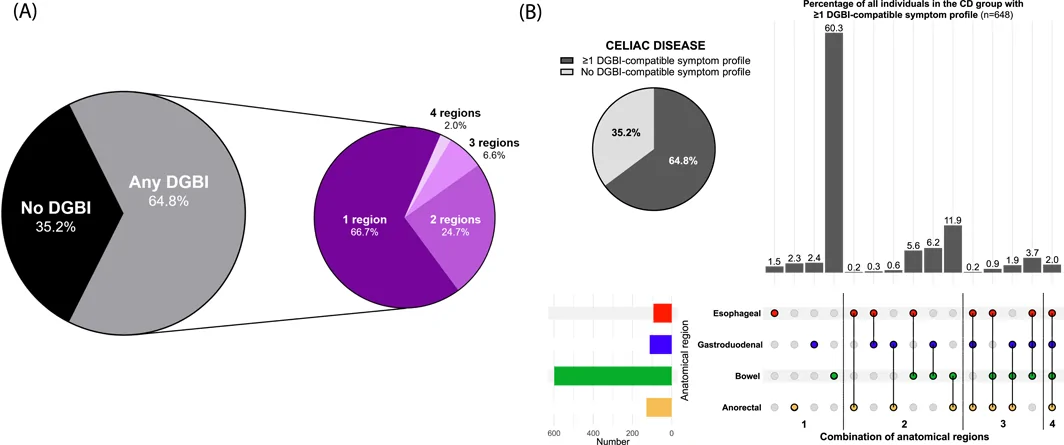
The number (A) and combinations (B) of overlapping anatomical regions of DGBI‐compatible symptom profiles in individuals with CD on a long‐term GFD. Explanation: 64.8% of the individuals with CD fulfiled the criteria for any DGBI‐compatible symptom profile. Within this group of individuals with any DGBI‐compatible symptom profile, 60.3% reported only symptoms compatible with a bowel DGBI and 11.9% reported symptoms compatible with both a bowel and anorectal DGBI but not of an oesophageal or gastroduodenal DGBI. The total number of individuals with CD reporting a bowel DGBI, regardless of the presence of DGBI‐compatible symptom profiles in other regions, was 599 (horizontal green bar). One individual with only functional biliary pain is not included in the upset diagram (Figure 1B). Supporting Information S1: Figure S2 shows the distribution in the reference group. CD; coeliac disease, DGBI; disorder of gut‐brain interaction, GFD; gluten‐free diet, *n*; number.

### 3.2 Association Between Coeliac Disease and DGBI‐Compatible Symptom Profiles

The prevalence of any DGBI‐compatible symptom profile was higher in individuals with CD than in the reference group (64.8% vs. 31.3%; adjusted OR (AOR) 3.2 (95% CI 2.6–3.8)), Table 3. The contributions of individual covariates in the multivariable logistic regression models are presented in Supporting Information S1: Table S1.

When investigating DGBI per anatomical region, the association between CD and DGBI‐compatible symptom profiles was present for bowel (AOR 3.2 (95% CI 2.6–3.8)) and anorectal DGBI (AOR 2.1 (95% CI 1.5–2.8)), and borderline present for oesophageal (AOR 1.3 (95% CI 1.0–1.9)) and gastroduodenal DGBI (AOR 1.4 (95% CI 1.0–1.9)).

Of the individual DGBIs, the strongest associations were observed for IBS (AOR 4.5 (95% CI 3.3–6.2)) and functional abdominal bloating and distension (AOR 3.9 (95% CI 2.4–6.4)). Furthermore, CD was linked to symptoms compatible with all subtypes of IBS: functional constipation, unspecified FBD, epigastric pain syndrome, and proctalgia fugax.

Sensitivity analyses applying stricter GFD adherence criteria (≥ 95% during the past year) and/or using an alternative reference group (NCV reference group) yielded similar results for the association between CD and having any DGBI‐compatible symptom profile, with AOR ranging 1.9–3.2 (Supporting Information S1: Tables 4‐6).

### 3.3 Factors Associated With DGBI‐Compatible Symptom Profiles in Individuals With CD

Medication use was more often reported in the group of individuals with CD and a DGBI‐compatible symptom profile compared with those without any of these symptom profiles. Among the medications assessed, over‐the‐counter medication to reduce pain (22.7%) and medication for constipation (14.8%) were most used by individuals with CD and a DGBI‐compatible symptom profile (Table 4).

**TABLE 4:** Medication use and surgical history of individuals with CD on a long‐term GFD with and without a DGBI‐compatible symptom profile.

	Overall CD cohort *n* = 1001	CD and any DGBI‐compatible symptom profile *n* = 649	CD and no DGBI‐compatible symptom profile *n* = 352	OR (95% CI)
Medication				
To reduce constipation, *n* (%)	112 (11.2%)	96 (14.8%)	16 (4.5%)	**3.6 (2.1–6.3)**
To reduce diarrhoea, *n* (%)	29 (2.9%)	23 (3.5%)	6 (1.7%)	2.1 (0.9–5.2)
To reduce nausea, *n* (%)	17 (1.7%)	15 (2.3%)	2 (0.6%)	4.1 (0.9–18.2)
To reduce pain: opioids (prescribed by a physician), *n* (%)	16 (1.6%)	13 (2.0%)	3 (0.9%)	2.4 (0.7–8.4)
To reduce pain: non‐opioids (prescribed by a physician), *n* (%)	64 (6.4%)	52 (8.0%)	12 (3.4%)	**2.5 (1.3–4.7)**
To reduce pain (not prescribed by a physician), *n* (%)	189 (18.9%)	147 (22.7%)	42 (11.9%)	**2.2 (1.5–3.1)**
To reduce gas or bloating, *n* (%)	45 (4.5%)	37 (5.7%)	8 (2.3%)	**2.6 (1.2–5.6)**
To reduce anxiety, *n* (%)	50 (5.0%)	44 (6.8%)	6 (1.7%)	**4.2 (1.8–9.9)**
To improve depression, *n* (%)	76 (7.6%)	60 (9.2%)	16 (4.5%)	**2.1 (1.2–3.8)**
To improve sleeping, *n* (%)	73 (7.3%)	59 (9.1%)	14 (4.0%)	**2.4 (1.3–4.4)**
Surgical history				
Cholecystectomy, *n* (%)	54 (5.4%)	47 (7.2%)	7 (2.0%)	**3.8 (1.7–8.6)**
Appendectomy, *n* (%)	107 (10.7%)	71 (10.9%)	36 (10.2%)	1.1 (0.7–1.6)
Hysterectomy^a^, *n* (%)	42 (5.3%)	33 (6.0%)	9 (3.7%)	1.6 (0.8–3.5)

*Note:* Bold values indicate ORs that do not cross one.

Abbreviations: CD, coeliac disease; DGBI, disorder of gut‐brain interaction; *n*, number; OR, odds ratio.

^a^ calculated as percentage of female participants (total *n* = 794; *n* = 518 included in the “any DGBI‐compatible symptom profile” group and *n* = 234 in the “no DGBI‐compatible symptom profile” group).

Psychological distress levels were higher among individuals with CD fulfiling the criteria for any DGBI compared with those who did not fulfill DGBI criteria (mean PHQ‐4: 2.8 vs. 1.5; SMD 0.48 (95% CI 0.35–0.61)). Similarly, non‐GI somatic symptom severity was higher in those with DGBI‐compatible symptom profiles (mean PHQ‐12: 6.9 vs. 4.3; SMD 0.78 (95% CI 0.65–0.92)). Both physical and mental health‐related QOL were lower in individuals with CD and a DGBI‐compatible symptom profile (mean PROMIS‐10 physical health: 13.8 vs. 16.0; SMD 0.80 (95% CI 0.67–0.93); mean PROMIS‐10 mental health: 13.2 versus 14.9; SMD 0.53 (95% CI 0.40–0.66). The levels of psychological distress and non‐GI somatic symptom severity were higher and levels of health‐related QOL lower in individuals with DGBI‐compatible symptom profiles in multiple anatomical regions (Figure 2A–D). A similar association was observed in the reference group, with higher levels of psychological distress and non‐GI somatic symptom severity and lower health‐related QOL associated with the increasing number of anatomical regions involved.

**FIGURE 2:**
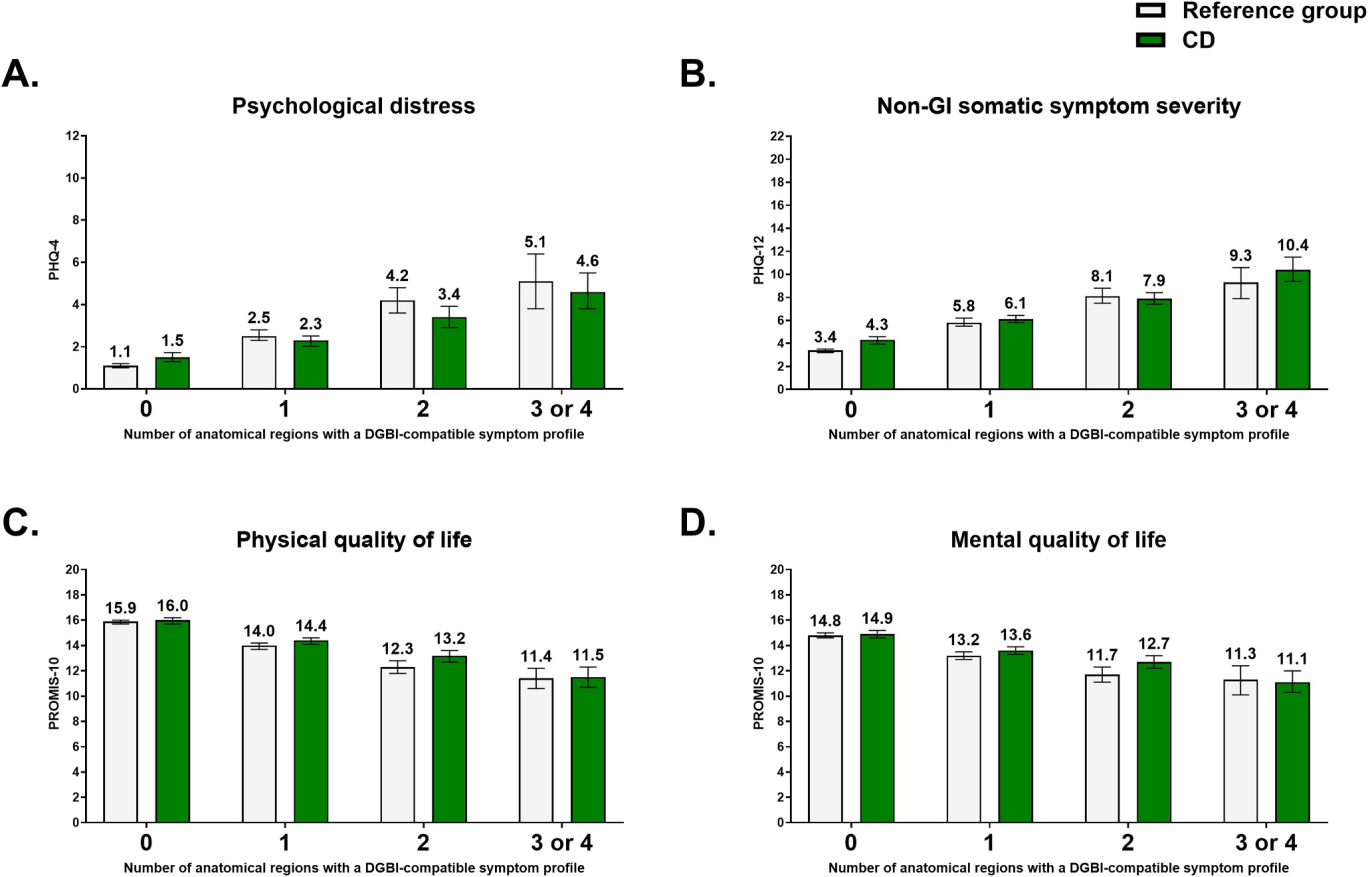
Mean levels of (A) psychological distress, (B) non‐GI somatic symptom severity, and (C) physical and (D) mental quality of life in individuals with CD and the reference group, stratified by the number of anatomical regions with a DGBI‐compatible symptom profile. The number of individuals with 0, 1, 2, and 3 or 4 anatomical regions with a DGBI‐compatible symptom profile were respectively *n* = 353, *n* = 432, *n* = 160, and *n* = 56 in the CD group and *n* = 1,304, *n* = 428, *n* = 125, and *n* = 39 in the reference group. CD; coeliac disease, DGBI; disorder of gut‐brain interaction, GI; gastrointestinal.

## 4 Discussion

This study shows that DGBI‐compatible symptom profiles are highly prevalent among individuals with CD despite adhering to a long‐term GFD, with higher prevalence rates in females and younger individuals. The observed association between CD and DGBI‐compatible symptom profiles held true after adjustment for multiple confounding factors, including sex, age, psychological distress, and non‐GI somatic symptom severity. Moreover, within the CD cohort, the presence of a DGBI‐compatible symptom profile was associated with higher levels of psychological distress, non‐GI somatic symptom severity, and a reduced health‐related QOL. Notably, the associations between having a DGBI‐compatible symptom profile and these factors were comparable with those observed in individuals without CD.

Although data are available about the presence of persistent symptoms in CD, the prevalence of persistent symptoms in the context of DGBI in patients with CD is understudied, except for IBS. To our knowledge, only two studies investigated the presence of all DGBI‐compatible symptom profiles in patients with CD on a GFD, resulting in a prevalence of 51%–65% of any DGBI [9, 27], which is in line with the current study. The prevalence of individual DGBI investigated in CD on a GFD is 8%–38% for IBS, 5%–14% for functional dyspepsia, and 5%–21% for functional abdominal bloating and distension [14, 27, 28, 29]. Notably, the prevalence of bowel DGBI in individuals with CD on a GFD is comparable with the prevalence in quiescent IBD, in which DGBI are more extensively studied [14, 27, 30]. A previously observed high prevalence and strong association between CD and Rome IV faecal incontinence could not be reproduced in the current study [27].

It should be noted that heterogeneity in the study design likely contributes to the large variation in reported prevalence estimates of persistent symptoms and DGBI in patients with CD on a long‐term GFD [5, 9, 28, 29, 31, 32, 33]. In our study, DGBI prevalence may have been overestimated due to selection bias, as recruitment through the patient society NCV and advertisement as a health survey may have preferentially attracted symptomatic individuals. However, NCV membership is routinely recommended by the Dutch CD guideline [34], likely making its members more representative of the broader CD population than members of other patient societies. To limit selection bias, the survey was not explicitly presented as assessing DGBI or (persistent) GI symptoms in both the CD and reference groups. Data were available from 1001 of 16,119 adult NCV members (6.2%), although this response rate should be interpreted in context, as non‐members could also participate and not all NCV members have CD.

Other factors contributing to the heterogeneity across studies include differences in recruitment strategy, assessment of GFD adherence or remission, and definitions of persistent symptoms, IBS, and DGBI. In a recent Swedish population‐based registry study [28], 2.7% of patients with CD were diagnosed with IBS during an average follow‐up of 11.1 years, compared with 18.2% in our cohort. This difference could partly be explained by the use of International Classification of Diseases codes in the Swedish study, as IBS may not be separately coded in patients with an existing CD diagnosis despite compatible persistent symptoms. Systematic symptom‐based assessment, such as the use of the Rome V diagnostic questionnaire, may therefore better reflect the presence of DGBI‐compatible symptom profiles in treated CD patients than registry‐based approaches.

We observed that several factors associated with fulfiling DGBI‐compatible symptom profiles in patients with CD were similar to those with DGBI in the general population. These include a higher prevalence among females and younger individuals, a similar distribution of DGBI overlap across anatomical regions, and higher levels of psychological distress as well as lower health‐related QOL in individuals with a DGBI [11, 35]. This suggests that the mechanisms involved in DGBI may also play a role in persistent symptoms in CD. The higher levels of non‐GI somatic symptom severity observed in individuals with CD and a DGBI‐compatible symptom profile should be interpreted with caution, since this could reflect not only heightened awareness of bodily symptoms but also extra‐intestinal symptoms related to CD. Notably, CD‐specific factors, including time on a GFD and age at diagnosis, were not associated with the presence of a DGBI‐compatible symptom profile, which is consistent with previous literature [5].

Studies investigating the underlying pathophysiological mechanisms of a concomitant DGBI in individuals with CD remain limited. Available data suggest a role for central mechanisms, although the association between CD and DGBI‐compatible symptom profiles in the current study persists even after adjustment for psychological distress and non‐GI somatic symptom severity [9]. Other proposed mechanisms besides general ones involved in DGBI include ongoing mucosal immune activity and changes in the microbiome related to CD [36, 37, 38, 39]. Also, dietary modifications related to a GFD, including a reduced fibre intake, might contribute to symptoms such as constipation and bloating [31].

Although the current study did not explicitly assess if physicians treat DGBI in parallel to CD, our findings show that DGBI‐ or symptom‐directed therapies are used by a subset of patients with CD and a DGBI‐compatible symptom profile. In addition, medications to reduce symptoms of anxiety and depression, frequently comorbid with DGBI [40], are also more commonly used by patients with CD who fulfil the criteria for a DGBI.

Differentiating DGBI from persistent CD activity can be challenging but will likely become more important if pharmacological therapies become available to treat CD in the future [41, 42]. Therefore, there is a need for clinically relevant non‐invasive biomarkers to differentiate between these two conditions in patients with CD. Furthermore, there is a need for consensus on the definition of DGBI in individuals with organic GI diseases, which recently has been done for IBS‐like symptoms in IBD [43]. The evidence for the treatment of DGBI in patients with CD is sparse, although there is some evidence for a positive effect of, for example, probiotics and a diet low in fermentable oligo‐, di‐ and monosaccharides, and polyols [36, 44]. Furthermore, it is likely that the evidence for treatment strategies of individuals with a DGBI without a concomitant organic GI disease can be extrapolated to those with both CD and a DGBI.

This study has several strengths. The study included a large group of individuals with CD and a reference group to investigate the relationship between CD and all 22 individual Rome IV DGBI‐compatible symptom profiles. This made it possible to investigate for the first time the association between CD and DGBI‐compatible symptom profiles after correcting for important confounding factors in a CD cohort on a long‐term GFD. Quality assurance checks were used to exclude individuals with potentially unreliable data, and sensitivity analyses were performed to validate the most important findings.

A limitation of this study is the self‐reported questionnaire‐based design. Since it is known that the reliability of self‐reported diagnoses varies [45], we have used strict criteria, by including only CD individuals who indicated that they were diagnosed by a physician, based on serology and/or histology. In individuals who fulfiled the criteria for a DGBI‐compatible symptom profile, unintentional gluten exposure, persistent villous atrophy, and other causes of persistent symptoms could not be excluded as causes for persistent symptoms. However, reliable tools to assess long‐term strict dietary adherence are currently lacking and evaluation of persistent enteropathy requires an invasive upper endoscopy with duodenal biopsies, which is not feasible in large‐scale studies [46]. Moreover, the clinical relevance of subtle histological abnormalities in relation to persistent symptoms remains uncertain, with Marsh I (intra‐epithelial lymphocytosis) considered as CD in remission [47]. Ideally, future studies should include more objective assessments of GFD adherence and histological recovery. Another limitation is the previously discussed risk of selection‐bias and the cross‐sectional design of the study, which makes it impossible to evaluate if the symptoms have changed from diagnosis.

In conclusion, our findings suggest that comorbid DGBI can be present in patients with CD on a GFD and that individuals with CD appear to be at increased risk of developing DGBI compared with the general population. The presence of comorbid DGBI is relevant since it is associated with higher levels of psychological distress and reduced levels of health‐related QOL. Future research should focus on how to define and diagnose a DGBI in individuals with CD and other organic GI diseases, to develop non‐invasive biomarkers to differentiate between CD activity and DGBI, and to define the best timing and method to treat these DGBI in patients with CD.

## Funding

This study was partially funded by the Dutch Coeliac Society (NCV). The Rome Foundation Global Epidemiology Study was funded by research grants from Ironwood, Shire, Allergan, and Takeda. None of the RFGES funders were involved in the planning, design, implementation, statistical analyses, or any other aspect of the study, including preparation of the article or knowledge of its contents.

## Ethics Statement

This study was reviewed by the Dutch medical ethics review committee ‘METC Brabant’ and was determined to be outside the restrictions of the Medical Research Involving Human Subjects Act (WMO) (protocol number: NW2025‐37). The Rome Foundation Global Epidemiology Study internet survey was reviewed by the Biomedical Institutional Review Board of the University of North Carolina at Chapel Hill (United States of America) and was deemed exempt from their oversight due to the anonymity of the participants.

## Consent

An electronic consent form was signed by all participants.

## Conflicts of Interest

Tom van Gils received speaker’s fee from Takeda. Hans Törnblom is a part of advisory board in Cinclus Pharma, Dr Falk GmbH, Medifactia, PRO.MED.CS, Purpose Pharma and received speaker’s fee from Enterra Medical, Medifactia, and Viatris. Inês A. Trindade received consultancy fees from Pfizer Inc. and received speaker’s fee from the Portuguese Inflammatory Bowel Disease Study Group (GEDII). Magnus Simrén received unrestricted research grants from BioGaia and received speakers fee or consultancy from Tillotts, Takeda, Biocodex, Sanofi, Abbvie, Janssen Immunology, Pfizer, BioGaia, Renapharma, AlfaSigma, Mayoly and Bromatech. All other authors declare no conflicts of interest.

## Permission to Reproduced Material From Other Sources

The authors have nothing to report.

## Supporting information


Supporting Information S1

## Data Availability

The dataset generated and analysed during the current study is not publicly available due to confidentiality. The data are available to researchers under a data use agreement with the Dutch Coeliac Society or the Rome Foundation (RFGES dataset) on reasonable request.
