# Supplementary material for: Symptom Profiles Compatible With Disorders of Gut‐Brain Interaction in Individuals With Coeliac Disease on a Long‐Term Gluten‐Free Diet
Source: United European Gastroenterol J. 2026 Oct 16;14(8):e70311. doi: 10.1002/ueg2.70311 (PMC13675726; doi:10.1002/ueg2.70311)
Supplement: Supplementary file 1 [file UEG2-14-e70311-s001.docx]

**SUPPLEMENTARY MATERIAL**

**Supplementary Methods**

**Supplementary Tables**

**Supplementary Figures**

**Supplementary Methods**

**Supplementary Methods**

The internal consistency of the questionnaires listed below was evaluated using Cronbach´s alpha values. Except for the Dutch Coeliac Dietary Adherence Test, the alphas ranged between 0.71 and 0.90, indicating acceptable to excellent internal consistencies ^1^. **Supplementary Methods Table 1** shows the Cronbach´s alpha for each questionnaire in our cohort.

**Questionnaires**

*Psychological distress: PHQ-4*

The PHQ-4 is a validated questionnaire to screen the severity of psychological distress ^2^. The PHQ-4 includes 2 items related to anxiety and 2 items to depressive symptoms. Higher PHQ-4 scores, ranging from 0 to 12, indicate more severe psychological distress.

*Non-GI somatic symptom severity: PHQ-12*

The PHQ-12 is a questionnaire to measure the severity of non-GI somatic symptoms ^3^. The question regarding menstruation-related symptoms was removed based on the sex- and age-specific character of that question. Individuals rated how much they have been bothered by each of the 11 remaining non-GI somatic symptoms during the past 4 week (0=not at all, 1=a little, 2=a lot). These include back pain, joint pain, intercourse-related pain or discomfort, headaches, chest pain, dizziness, fainting spells, palpitations, shortness of breath, tiredness, and trouble sleeping. Higher PHQ-12 scores, ranging from 0 to 22, indicate more severe non-GI somatic symptoms, which is often used as a surrogate maker for heightened awareness of bodily symptoms.

*Health-related quality of life: PROMIS Global-10*

The PROMIS Global-10 is a validated questionnaire to assess global physical and mental health ^4, 5^. Both subscales include 4 questions. Higher subscale scores, ranging from 4 to 20, indicate higher health-related quality of life.

*Self-reported gluten-free diet (GFD) adherence*

Participants with CD were asked: “During the past year, what percentage of time do you estimate that you adhered to the GFD”? Response options were provided with 10% ranges (0%, 1-9%, 10-19%, 20-29%, etc), except for 90-94%, 95-99%, and 100% of the time.

*Coeliac Dietary Adherence Test (CDAT)*

The CDAT is a questionnaire intending to assess if someone with CD is optimally adherent to a GFD ^6^. The CDAT includes 7 items, with a total score ranging from 5 to 35, and with a score <13 indicating an optimal GFD adherence in individuals with CD. The original CDAT was developed and validated in US-English, but a Dutch version was not available. For the purpose of this study, we translated and psychometrically analyzed the Dutch-CDAT.

Before data collection, the CDAT was translated to Dutch by two translators and back-translated to English by one translator, all fluent in both Dutch and English. Adjustments to ensure alignment with the original version were performed by discussion with an independent person also fluent in both languages. After this, four Dutch individuals with CD completed the CDAT and provided feedback on the scale. Final adjustments to the questionnaire were then made considering the feedback.

Results showed that the internal consistency of Dutch-CDAT was not acceptable (Cronbach´s alpha = 0.38; values ≥0.70 are considered acceptable ^1^). Results regarding construct validity and the questionnaire´s structure were obtained using confirmatory factor analysis (CFA) (IBM Amos SPSS 30) ^7^. An initial CFA showed a very poor model fit and suggested the correlation between item 6 (“How important to your health are accidental gluten exposures?”) and item 7 (“Over the past 4 weeks, how many times have you eaten foods containing gluten on purpose?”). After this correlation was established, the model was rerun, but the model fit was still poor; χ^2^/df = 11.1 (should be <5); Comparative Fit Index = 0.77 (should be ≥0.95), Root Means Square Error of Approximation = 0.10 (should be <0.06) ^7, 8^. More details are shown in **Supplementary Methods Figure 1**. Based on the very poor reliability and lack of construct validity of the Dutch-CDAT, this questionnaire was considered psychometrically inadequate and therefore was not used in this study’s analyses.

|  | **Internal consistency (Cronbach’s alpha)** | | |
| --- | --- | --- | --- |
|  | **Coeliac disease** | **RFGES**  **reference group** | **NCV**  **reference group** |
| **PHQ-4** | 0.88 | 0.90 | 0.86 |
| **PHQ-12*** | 0.76 | 0.76 | 0.73 |
| **PROMIS-10 global physical health** | 0.78 | 0.76 | 0.71 |
| **PROMIS-10 global mental health** | 0.84 | 0.84 | 0.83 |
| **Supplementary Methods Table 1. Internal consistencies of the questionnaires.**  A Cronbach’s alpha´s ≥0.70 are considered as acceptable ^1^.  * Without the item that assesses menstruation-related symptoms. | | | |

|  | **Supplementary Methods Figure 1. Confirmatory Factor Analysis of the Dutch-CDAT** |
| --- | --- |

**REFERENCES SUPPLEMENTARY METHODS**

1.	Kalkbrenner MT. Choosing Between Cronbach’s Coefficient Alpha, McDonald’s Coefficient Omega, and Coefficient H: Confidence Intervals and the Advantages and Drawbacks of Interpretive Guidelines. Measurement and Evaluation in Counseling and Development. 2024;57(2):93-105.

2.	Kroenke K, Spitzer RL, Williams JB, et al. An ultra-brief screening scale for anxiety and depression: the PHQ-4. Psychosomatics. 2009;50(6):613-21. Epub 2009/12/10.

3.	Spiller RC, Humes DJ, Campbell E, et al. The Patient Health Questionnaire 12 Somatic Symptom scale as a predictor of symptom severity and consulting behaviour in patients with irritable bowel syndrome and symptomatic diverticular disease. Aliment Pharmacol Ther. 2010;32(6):811-20. Epub 2010/07/16.

4.	Available from: <https://www.codetechnology.com/blog/promis-global-10/>.

5.	Hays RD, Bjorner JB, Revicki DA, et al. Development of physical and mental health summary scores from the patient-reported outcomes measurement information system (PROMIS) global items. Qual Life Res. 2009;18(7):873-80. Epub 2009/06/23.

6.	Leffler DA, Dennis M, Edwards George JB, et al. A simple validated gluten-free diet adherence survey for adults with celiac disease. Clin Gastroenterol Hepatol. 2009;7(5):530-6, 6.e1-2. Epub 2009/03/10.

7.	Babyak MA, Green SB. Confirmatory factor analysis: an introduction for psychosomatic medicine researchers. Psychosom Med. 2010;72(6):587-97. Epub 2010/05/15.

8.	Hair JF, Tatham RL, Anderson RE, et al. Multivariate Data Analysis 5th ed: Prentice Hall; 1998.

**Supplementary Tables**

|  | **Coeliac disease**  n=1,001 | **NCV reference group**  n=193 |
| --- | --- | --- |
| **Age, median [IQR]** | 50 [35 - 64] | 48 [38 - 62] |
| **Sex** |  |  |
| *Female, n (%)* | 794 (79.3%) | 127 (65.8%) |
| *Male, n(%)* | 204 (20.4%) | 66 (34.2%) |
| *Other, n (%)* | 1 (0.1) | 0 (0%) |
| *Unknown, n (%)* | 2 (0.2) | 0 (0%) |
| **Completed higher-level professional or university-level education, n (%)** | 635 (63.4%) | 143 (74.1%) |
| **Non-GI somatic symptom severity (PHQ-12), median [IQR]** | 6 [3 - 8] | 4 [2 -7] |
| **Psychological distress (PHQ-4), median [IQR]** | 2 [0 - 4] | 1 [0 - 3] |
| **Parent, brother, or sister with CD, n (%)** | 229 (22.9%) | 25 (13.0%) |

**Supplementary Table 1. Characteristics of the CD and NCV reference group**

CD, coeliac disease; IQR; interquartile range, n; number, OR; odds ratio.

|  | **Reference group** | **OR (95%CI)** |
| --- | --- | --- |
| **Total reference group**  n=1,896 | 31.3 (29.2-33.4) | - |
| **Sex** |  |  |
| Male  n=932 | 21.8 (19.2-24.5) | reference |
| Female  n=964 | 40.5 (37.4-43.6) | **2.4 (2.0-3.0)** |
| **Age** |  |  |
| 18-34 years  n=615 | 38.4 (34.6-42.3) | **2.3 (1.7-3.2)** |
| 35-49 years  n=387 | 34.1 (29.6-39.0) | **1.9 (1.4-2.7)** |
| 50-64 years  n=532 | 28.0 (24.4-32.0) | **1.5 (1.1-2.0)** |
| ≥65 years  n=362 | 21.0 (17.1-25.5) | reference |

**Supplementary Table 2. Prevalence of DGBI-compatible symptom profiles in subgroups of the RFGES reference group.**

n; number, OR; odds ratio

|  | **Dependent variable:**  **Having any DGBI-compatible symptom profile** | | |
| --- | --- | --- | --- |
|  | **Crude OR**  (95% CI) | **Adjusted OR**  **Model 1**  (95% CI) | **Adjusted OR**  **Model 2**  (95% CI) |
| **Coeliac disease,**  ***(RFGES reference group = 0, coeliac disease = 1)*** | 4.05 (3.45 – 4.76) | 3.59 (3.02 – 4.26) | 3.16 (2.62 – 3.81) |
| **Age*,* years, *continuous*** | 0.99 (0.98 – 0.99) | 0.99 (0.98 – 0.99) | 1.00 (0.99 – 1.00) |
| **Sex, *(male=0, female=1)*** | 3.21 (2.73 – 3.77) | 2.24 ( 1.89 – 2.67) | 1.70 (1.40 – 2.05) |
| **Psychological distress (PHQ-4)*, continuous*** | 1.34 (1.29 – 1.38) | - | 1.13 (1.09 – 1.18) |
| **Non-GI somatic symptom severity (PHQ-12)*, continuous*** | 1.36 (1.32 – 1.39) | - | 1.26 (1.22 – 1.30) |

**Supplementary Table 3. Associations between variables (CD and covariates) included in the multivariable logistic regression models and having any DGBI-compatible symptom profile (dependent variable).**

GI; gastrointestinal, OR; odds ratio

|  | **Celiac disease**  **Self-reported GFD adherence ≥95%**  n=947 | **RFGES**  **Reference group**  n=1,896 | **Crude OR**  **(95%CI)** | **AOR**  **(95%CI)**  *Adjusted for sex and age* | **AOR**  **(95%CI)**  *Adjusted for sex, age, non-GI somatic symptom severity, and psychological distress* |
| --- | --- | --- | --- | --- | --- |
| **Any DGBI** | 614 (64.8%) | 593 (31.3%) | **4.1 (3.4 – 4.8)** | **3.6 (3.0 – 4.3)** | **3.2 (2.6 – 3.9)** |
| **Any esophageal DGBI** | 84 (8.9%) | 92 (4.9%) | **1.9 (1.4 – 2.6)** | **1.7 (1.2 – 2.3)** | 1.3 (0.9 – 1.8) |
| **Any gastroduodenal DGBI** | 108 (11.4%) | 116 (6.1%) | **2.0 (1.5 – 2.6)** | **1.9 (1.4 – 2.6)** | **1.5 (1.1 – 2.1)** |
| *Functional dyspepsia* | 68 (7.2%) | 78 (4.1%) | **1.8 (1.3 – 2.5)** | **1.8 (1.2 – 2.5)** | 1.3 (0.9 – 1.9) |
| **Any bowel DGBI** | 565 (59.7%) | 511 (27.0%) | **4.0 (3.4 – 4.7)** | **3.6 (3.0 – 4.2)** | **3.2 (2.6 – 3.8)** |
| *IBS* | 173 (18.3%) | 73 (3.9%) | **5.6 (4.2 – 7.4)** | **5.2 (3.9 – 7.1)** | **4.7 (3.4 – 6.4)** |
| *Functional constipation* | 154 (16.3%) | 178 (9.4%) | **1.9 (1.5 – 2.4)** | **1.6 (1.2 – 2.0)** | **1.4 (1.1 – 1.8)** |
| *Functional diarrhea* | 52 (5.5%) | 64 (3.4%) | **1.7 (1.1 – 2.4)** | 1.5 (1.0 – 2.2) | 1.3 (0.9 – 2.0) |
| *Functional bloating/distension* | 57 (6.0%) | 26 (1.4%) | **4.6 (2.9 – 7.4)** | **4.2 (2.5 – 6.9)** | **3.9 (2.3 – 6.4)** |
| **Any anorectal DGBI** | 124 (13.1%) | 84 (4.4%) | **3.3 (2.4 – 4.3)** | **2.7 (2.0 – 3.6)** | **2.1 (1.5 – 2.9)** |

**Supplementary Table 4. The association between having CD on a long-term GFD and fulfilling criteria for a Rome IV DGBI, including only individuals with CD and a self-reported GFD adherence of ≥95% during the past year and the RFGES reference group.**

*Only the most common individual DGBI, except FBD unspecified and proctalgia fugax, are included as individual DGBI-compatible symptom profile.*

AOR; adjusted odds ratio, CD; coeliac disease, DGBI; disorder of gut-brain interaction, n; number, FBD; functional bowel disorder, GFD; gluten-free diet, IBS; irritable bowel syndrome, n; number, OR; odds ratio, RFGES; Rome Foundation Global Epidemiology Study

|  | **Coeliac disease**  **Self-reported GFD adherence ≥95%**  n=947 | **NCV reference group**  n=193 | **Crude OR**  **(95%CI)** | **AOR**  **(95%CI)**  *Adjusted for sex and age* | **AOR**  **(95%CI)**  *Adjusted for sex, age, non-GI somatic symptom severity, and psychological distress* |
| --- | --- | --- | --- | --- | --- |
| **Any DGBI** | 614 (64.8%) | 84 (43.5%) | **2.4 (1.7 – 3.3)** | **2.2 (1.6 – 3.1)** | **1.9 (1.4 – 2.7)** |
| **Any esophageal DGBI** | 84 (8.9%) | 4 (2.1%) | **4.6 (1.7 – 12.7)** | **4.4 (1.6 – 12.1)** | **3.4 (1.2 – 9.5)** |
| **Any gastroduodenal DGBI** | 108 (11.4%) | 8 (4.1%) | **3.0 (1.4 – 6.2)** | **2.7 (1.3 – 5.7)** | 2.1 (1.0 – 4.5) |
| *Functional dyspepsia* | 68 (7.2%) | 7 (3.6%) | 2.1 (0.9 – 4.5) | 1.8 (0.8 – 4.1) | 1.3 (0.6 – 2.9) |
| **Any bowel DGBI** | 565 (59.7%) | 81 (42.0%) | **2.0 (1.5 – 2.8)** | **1.9 (1.4 – 2.6)** | **1.6 (1.2 – 2.3)** |
| *IBS* | 173 (18.3%) | 16 (8.3%) | **2.5 (1.4 – 4.2)** | **2.2 (1.3 – 3.8)** | **1.9 (1.1 – 3.3)** |
| *Functional constipation* | 154 (16.3%) | 25 (13.0%) | 1.3 (0.8 – 2.1) | 1.2 (0.7 – 1.8) | 1.1 (0.7 – 1.8) |
| *Functional diarrhea* | 52 (5.5%) | 5 (2.6%) | 2.2 (0.9 – 5.5) | 2.1 (0.8 – 5.5) | 2.1 (0.8 – 5.5) |
| *Functional bloating/distension* | 57 (6.0%) | 11 (5.7%) | 1.1 (0.5 – 2.1) | 1.0 (0.5 – 2.0) | 0.9 (0.5 – 1.8) |
| **Any anorectal DGBI** | 124 (13.1%) | 14 (7.3%) | **1.9 (1.1 – 3.4)** | 1.7 (1.0 – 3.1) | 1.4 (0.8 – 2.6) |

**Supplementary Table 5. The association between having CD on a long-term GFD and fulfilling criteria for a Rome IV DGBI, including only individuals with CD and a self-reported GFD adherence of ≥95% during the past year and the NCV reference group.**

*Only the most common individual DGBI, except FBD unspecified and proctalgia fugax, are included as individual DGBI-compatible symptom profile.*

AOR; adjusted odds ratio, CD; coeliac disease, DGBI; disorder of gut-brain interaction, n; number, FBD; functional bowel disorder, GFD; gluten-free diet, IBS; irritable bowel syndrome, n; number, NCV; Dutch Coeliac Society, OR; odds ratio

|  | **Coeliac disease**  n=1,001 | **NCV reference group**  n=193 | **Crude OR**  **(95%CI)** | **AOR**  **(95%CI)**  *Adjusted for sex and age* | **AOR**  **(95%CI)**  *Adjusted for sex, age, non-GI somatic symptom severity, and psychological distress* |
| --- | --- | --- | --- | --- | --- |
| **Any DGBI** | 649 (64.8%) | 84 (43.5%) | **2.4 (1.8 – 3.3)** | **2.2 (1.6 – 3.0)** | **1.9 (1.4 – 2.7)** |
| **Any esophageal DGBI** | 93 (9.3%) | 4 (2.1%) | **4.8 (1.8 – 13.3)** | **4.6 (1.7 – 12.6)** | **3.6 (1.3 – 10.0)** |
| **Any gastroduodenal DGBI** | 112 (11.2%) | 8 (4.1%) | **2.9 (1.4 – 6.1)** | **2.7 (1.3 – 5.6)** | 2.0 (0.9 – 4.3) |
| *Functional dyspepsia* | 71 (7.1%) | 7 (3.6%) | 2.0 (0.9 – 4.5) | 1.8 (0.8 – 4.0) | 1.2 (0.5 – 2.8) |
| **Any bowel DGBI** | 204 (51.9%) | 81 (42.0%) | **2.1 (1.5 – 2.8)** | **1.9 (1.4 – 2.6)** | **1.6 (1.2 – 2.3)** |
| *IBS* | 182 (18.2%) | 16 (8.3%) | **2.5 (1.4 – 4.2)** | **2.2 (1.3 – 3.8)** | **1.8 (1.0 – 3.2)** |
| *Functional constipation* | 162 (16.2%) | 25 (13.0%) | 1.3 (0.8 – 2.0) | 1.2 (0.7 – 1.8) | 1.1 (0.7 – 1.8) |
| *Functional diarrhea* | 55 (5.5%) | 5 (2.6%) | 2.2 (0.9 – 5.5) | 2.2 (0.9 – 5.6) | 2.2 (0.8 – 5.5) |
| *Functional bloating/distension* | 61 (6.1%) | 11 (5.7%) | 1.1 (0.6 – 2.1) | 1.0 (0.5 – 2.0) | 0.9 (0.5 – 1.9) |
| **Any anorectal DGBI** | 129 (12.9%) | 14 (7.3%) | **1.9 (1.1 – 3.4)** | 1.7 (1.0 – 3.0) | 1.4 (0.8 – 2.5) |

**Supplementary Table 6. The association between having CD on a long-term GFD and fulfilling criteria for a Rome IV DGBI, including all individuals with CD and ≥1 year on a GFD and the NCV reference group.**

*Only the most common individual DGBI, except FBD unspecified and proctalgia fugax, are included as individual DGBI-compatible symptom profile.*

AOR; adjusted odds ratio, CD; coeliac disease, DGBI; disorder of gut-brain interaction, n; number, FBD; functional bowel disorder, GFD; gluten-free diet, IBS; irritable bowel syndrome, n; number, NCV; Dutch Coeliac Society, OR; odds ratio

**Supplementary Figures**

**Supplementary Figure 1. Participant flow diagram**

*RFGES data cleaning is not shown in this study flow chart.*

*CD; coeliac disease, GI; gastrointestinal, IBD; inflammatory bowel disease, n; number, NCGS; non-coeliac gluten sensitivity; NCV; Dutch Coeliac Society, RFGES; Rome Foundation Global Epidemiology Study*

| **A.** |  | **B.** |  |
| --- | --- | --- | --- |
| **Supplementary Figure 2. The number (A) and combinations (B) of overlapping anatomical regions of DGBI-compatible symptom profiles in the reference group.**  *Explanation: 31.1% of the individuals in the reference group fulfilled the criteria for any DGBI-compatible symptom profile. Within this group of individuals with any DGBI-compatible symptom profile, 61.0% reported only symptoms compatible with a bowel DGBI and 7.4% reported symptoms compatible with both a bowel and gastroduodenal, but not of a esophageal or anorectal DGBI. The total number of individuals with CD reporting a bowel DGBI, regardless of the presence of DGBI-compatible symptom profiles in other regions, was 511 (horizontal green bar). One individual with only functional biliary pain is not included in the upset diagram.*  DGBI; disorder of gut-brain interaction, GFD; gluten-free diet, n; number | | | |
